# Supplementary material for: Hippo Signaling Pathway Activation during SARS-CoV-2 Infection Contributes to Host Antiviral Response
Source: bioRxiv. 2022 Apr 8:2022.04.07.487520. Preprint. [Version 1] doi: 10.1101/2022.04.07.487520 (PMC9016637; doi:10.1101/2022.04.07.487520)
Supplement: 1 [file NIHPP2022.04.07.487520V1-supplement-1.pdf]

bioRxiv preprint doi: https://doi.org/10.1101/2022.04.07.487520; this version posted April 8, 2022. The copyright holder for this preprint (which was not certified by peer review) is the author/funder, who has granted bioRxiv a license to display the preprint in perpetuity. It is made available under aCC-BY 4.0 International license.

SUPPLEMENTARY FIGURES

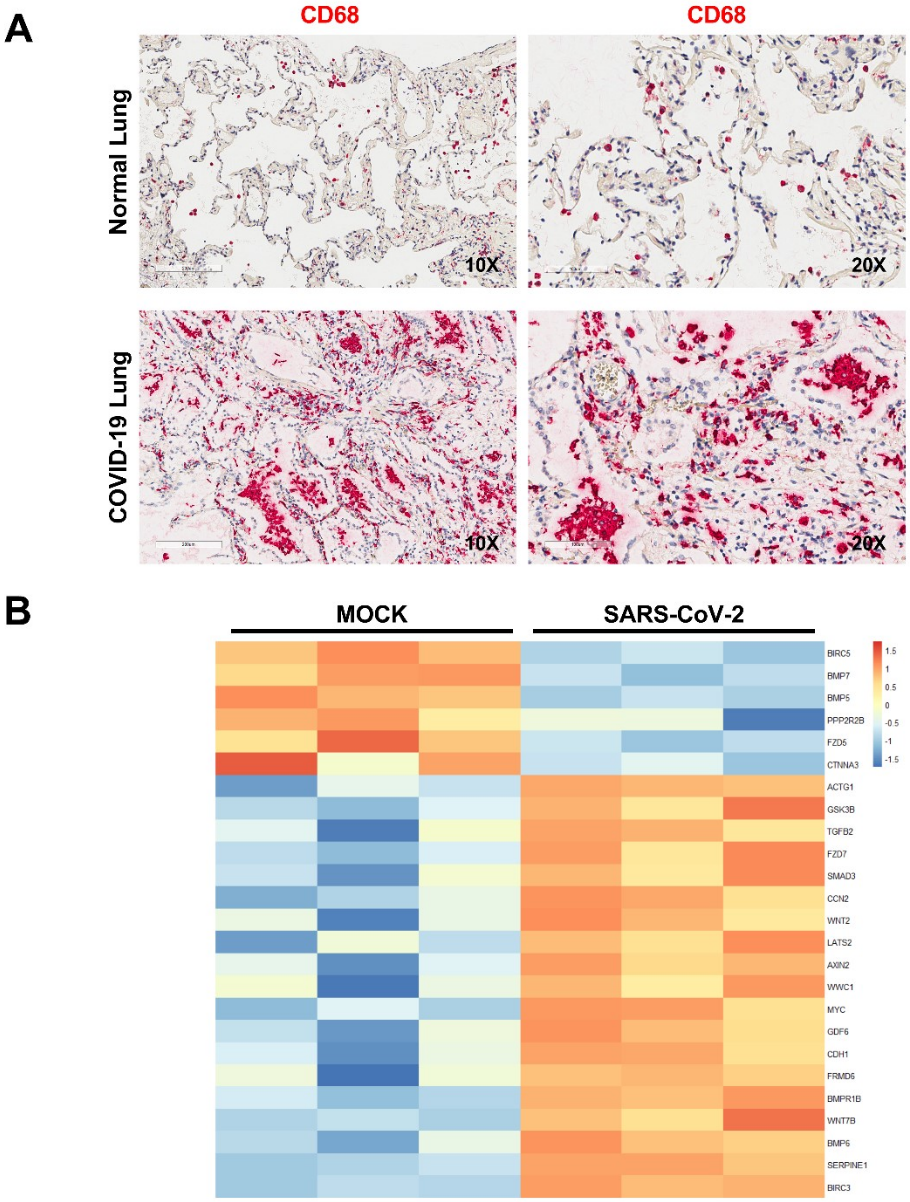

Supplementary Figure 1. (A) Immunohistochemistry of COVID-19 lung autopsy tissue shows high level of CD68 positive inflammatory cells (red). Images are obtained at 10x and 20x magnifications. (B) Transcriptome analysis of control and SARS-CoV-2 infected human induced PSC-CMs at 3 dpi. Heatmap depicting *Z* scores as expression levels of the 25 differentially expressed genes (p <0.01) involved in Hippo signaling pathway. Blue and red colors represent downregulated and upregulated genes, respectively. The gene expression data was retrieved at Gene Expression Omnibus with accession number GSE150392.

bioRxiv preprint doi: https://doi.org/10.1101/2022.04.07.487520; this version posted April 8, 2022. The copyright holder for this preprint (which was not certified by peer review) is the author/funder, who has granted bioRxiv a license to display the preprint in perpetuity. It is made available under aCC-BY 4.0 International license.

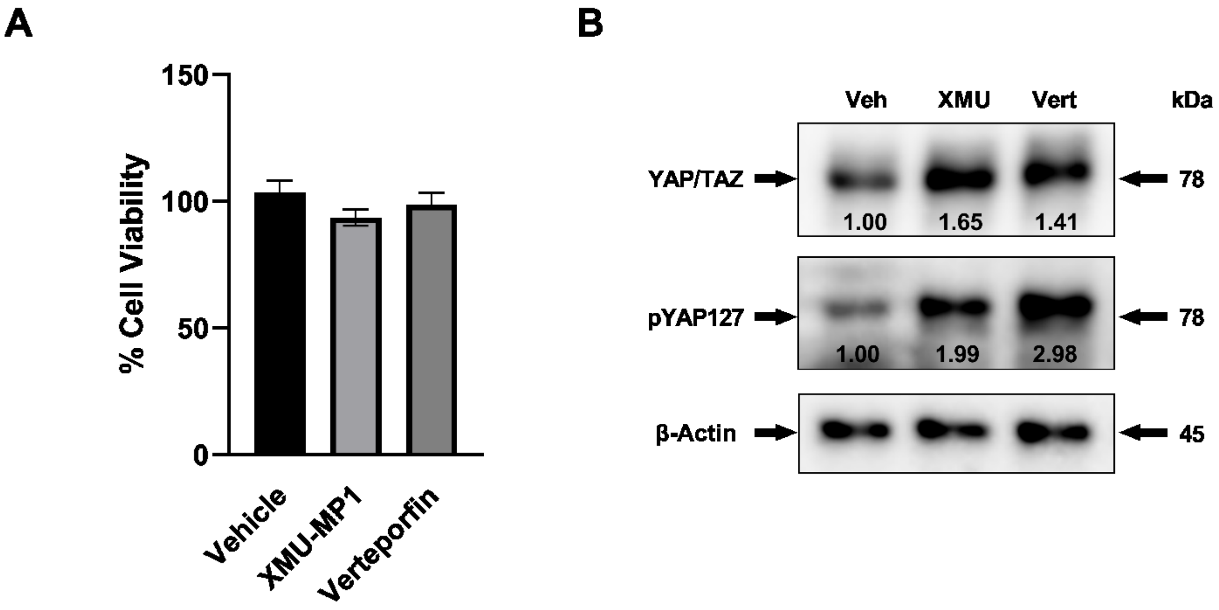

Supplementary Figure 2. (A) Graph shows the percent cytotoxicity of Calu-3 cells 72 hours post-treatment with DMSO (Vehicle), XMU-MP1 (10μM), and Verteporfin (1 μM). CellTiter-Glo Luminescent Cell Viability Assay was performed as per the manufacturer (Promega, USA) recommendation. (B) Western blot analysis shows total and phosphorylated YAP at 72 hours-post treatment with indirect and direct acting inhibitors, XMU-MP-1 and Verteporfin, respectively. Note: XMU-MP-1 treatment enhances YAP/TAZ level compared to vehicle, whereas Verteporfin increases phosphorylated YAP (S127) levels. Representative data from two independent experiments is shown.

SUPPLEMENTAL TABLES

Supplementary Table 1: Reagents or resources used in this study.

REAGENT/RESOURCE	SOURCE	IDENTIFIER
**Antibodies**		
Monoclonal anti-SARS-CoV S protein (Similar to 240C) antibody	BEI Resources Repository	Cat#NR-616
Polyclonal anti-SARS coronavirus (antiserum)	BEI Resources Repository	Cat#NR-10361
TBK1/NAK (D1B4)	Cell Signaling Technology	Cat#3504S
Phospho-TBK1/NAK (Ser172)	Cell Signaling Technology	Cat#5483S
YAP/TAZ (D24E4)	Cell Signaling Technology	Cat# 8418S
Phospho-YAP (Ser127)	Cell Signaling Technology	Cat#13008S
COVID V-nCoV2019-S probe	ACD	Cat#: 848568

bioRxiv preprint doi: https://doi.org/10.1101/2022.04.07.487520; this version posted April 8, 2022. The copyright holder for this preprint (which was not certified by peer review) is the author/funder, who has granted bioRxiv a license to display the preprint in perpetuity. It is made available under aCC-BY 4.0 International license.

AC TUBULIN	Cell Signaling Technology	Cat#mAb#5335
MUC5AC Mouse	Invitrogen	Cat#MA512178
Anti-Cardiac Troponin T antibody	Abcam	Cat#ab45932
Cleaved Caspase 3	Cell Signaling Technology	Cat# 9661S
Cleaved caspase-3 rabbit monoclonal antibody, clone D175	Cell Signaling	Cat#9661S
Goat anti-Mouse IgG (H+L) Cross-Adsorbed Secondary Antibody, Alexa Fluor 555	Thermo Fisher Scientific	Cat#A21422
IgG (H+L) Cross-Adsorbed Goat anti-Rabbit, Alexa Fluor 488, Invitrogen	Thermo Fisher Scientific	Cat#A11008
IgG (H+L) Cross-Adsorbed Goat anti-Human, Alexa Fluor 488, Invitrogen	Thermo Fisher Scientific	Cat#A11013
Goat anti-Guinea Pig IgG (H+L) Highly Cross-Adsorbed Secondary Antibody, Alexa Fluor 488	Thermo Fisher Scientific	Cat#A11073
Monoclonal Anti-Beta-Actin, Clone AC-74 produced in mouse	MilliporeSigma	Cat#A2228
Phospho-Stat1 (Tyr701) (58D6) Rabbit mAb	Cell Signaling	Cat#9167S
Stat1 (D1K9Y) Rabbit mAb	Cell Signaling	Cat#14994
CD68	Dako	Cat#m0876
**Bacterial and Virus Strains**		
SARS-Related Coronavirus 2 (SARS-CoV-2), Isolate USA-WA1/2020	BEI Resources Repository	Cat#NR-52281
SARS-Related Coronavirus 2, Isolate hCoV-19/USA/MD-HP05647/2021 (Lineage B.1.617.2; Delta variant),	BEI Resources Repository	Cat#NR-55672
SHCLNG MISSION shRNA YAP1 Bacterial Clone	Sigma-Aldrich	Cat# TRCN0000107267
SHCLNG MISSION shRNA LATS1 Bacterial Clone	Sigma-Aldrich	Cat# TRCN0000001779
**Chemicals, Peptides, and Recombinant Proteins**		
Regular Fetal Bovine Serum	Corning	Cat#35010CV
Eagle's Minimum Essential Medium (MEM)	Corning	Cat#10009CV
Penicillin-Streptomycin (10,000 U/mL)	Gibco	Cat#15140122
L-Glutamine (200 mM)	Gibco	Cat#25030081
Puromycin Dihydrochloride	Gibco	Cat#A1113803
PneumaCult™-ALI Medium	STEMCELL Technologies	Cat#05021
AR9 BUFFER, 10X	Akoya Biosciences	Cat#AR9001KT
Dimethyl sulfoxide	MilliporeSigma	Cat#D2650
RPMI 1640	Thermo Fisher Scientific	Cat#11875093
B27 supplement with insulin	Thermo Fisher Scientific	Cat#17504044
Methanol (Histological)	Thermo Fisher Scientific	Cat#A433P4
16% Paraformaldehyde (formaldehyde) aqueous solution	Electron Microscopy Sciences	Cat#15710
Dulbecco's Phosphate-Buffered Salt Solution 1X	Corning	Cat#21030CV
Perm/Wash Buffer	BD Biosciences	Cat#554723

bioRxiv preprint doi: https://doi.org/10.1101/2022.04.07.487520; this version posted April 8, 2022. The copyright holder for this preprint (which was not certified by peer review) is the author/funder, who has granted bioRxiv a license to display the preprint in perpetuity. It is made available under aCC-BY 4.0 International license.

DAPI (4',6-Diamidino-2-Phenylindole, Dihydrochloride)	Thermo Fisher Scientific	Cat#D1306
SuperBlock™ (PBS) Blocking Buffer	Thermo Fisher Scientific	Cat#37515
Bovine Serum Albumin	MilliporeSigma	Cat#A9418
Normal Donkey Serum	Jackson ImmunoResearch	Cat#017-000-121
Normal Goat Serum	Cell Signaling	Cat#5425S
Triton-X 100	MilliporeSigma	Cat#T9284
XMU-MP-1	MilliporeSigma	Cat# SML2233-5MG
Verteporfin	MilliporeSigma	Cat#SML0534-5MG
**Commercial Assays**		
Bond Polymer Refine Detection Kit	Leica Microsystems	Cat#DS9800
CellTiter 96® Non-Radioactive Cell Proliferation Assay (MTT)	Promega	Cat#G4000
**Experimental Models: Cell Lines**		
VERO C1008 [Vero 76, clone E6, Vero E6]	ATCC	Cat#CRL-158
Calu-3	ATCC	Cat#HTB-55
hPSC derived cardiomyocyte	University of California, Los Angeles (Li et al., 2021)	N/A
Normal human bronchial epithelial cells	Lonza	N/A
**Deposited Data**		
RNA-Seq of Human iPSC-cardiomyocytes infected with SARS-CoV-2	Gene Expression Omnibus	Accession Number: GSE150392
**Software and Algorithms**		
GraphPad Prism 8	GraphPad	N/A
Multi-Point Tool (Cell Counter)	ImageJ	N/A
